# From targets to ripples: tracing the process of developing a community capacity building appraisal tool with remote Australian indigenous communities to tackle food security

**DOI:** 10.1186/1471-2458-14-914

**Published:** 2014-09-04

**Authors:** Julie Brimblecombe, Christel van den Boogaard, Jan Ritchie, Ross Bailie, John Coveney, Selma Liberato

**Affiliations:** Menzies School of Health Research, Royal Darwin Hospital, PO Box 41096, Darwin, Northern Territory, 0811 Australia; University of New South Wales, Sydney, New South Wales Australia; Flinders University, Adelaide, South Australia Australia

**Keywords:** Community capacity development, Appraisal, Multi-sectoral, Indigenous health, Food security

## Abstract

**Background:**

The issue of food security is complex and requires capacity for often-unrelated groups to work together. We sought to assess the relevance and meaning of a commonly used set of community capacity development constructs in the context of remote Indigenous Australia and through this propose a model to support capacity.

**Methods:**

The assessment was conducted with four communities and took place over five steps that involved: (i) test of clarity of construct meaning; (ii) inductive derivation of community capacity constructs; (iii) application of these constructs to the capacity of community multi-sector food-interest groups; (iv) a cross-check of these constructs and their meanings to literature-derived constructs, and; (v) achieving consensus on tool constructs. Data were collected over a three-year period (2010–2012) that involved two on-site visits to one community, and two urban-based workshops. These data were augmented by food-interest group meeting minutes and reports.

**Results:**

Eleven community capacity development constructs were included in the proposed model: community ownership, building on strengths, strong leadership and voice, making decisions together, strong partnerships, opportunities for learning and skill development, way of working, getting together the things you need, good strong communication, sharing the true story, and continuing the process and passing on to the next generation.

The constructs derived from the literature and commonly used to appraise community capacity development were well accepted and could be used to identify areas needing strengthening. The specifics of each construct however differed from those derived from the literature yet were similar across the four communities and had particular meaning for those involved. The involvement of elders and communication with the wider community seemed paramount to forming a solid foundation on which capacity could be further developed.

**Conclusion:**

This study explored an approach for ascribing context specific meanings to a set of capacity development constructs and an effective visual appraisal tool. An approach to tackling food security in the remote Indigenous context where community capacity goals are considered in parallel with outcome goals, or at least as incremental goals along the way, may well help to lay a more solid foundation for improved service practice and program sustainability.

**Electronic supplementary material:**

The online version of this article (doi:10.1186/1471-2458-14-914) contains supplementary material, which is available to authorized users.

## Background

Food insecurity, which relates to food availability, access and use, contributes to the disadvantage experienced by Indigenous Australians [1] and an excessive burden of preventable chronic disease [2] and undernutrition in children [1]. Many factors relating to the physical, economic, political and socio-cultural environments contribute to food insecurity [1] making it a highly complex and dynamic issue with no simple solution [3]. Approaches to address food security that are multi-sectoral, involve the whole-of-society, and are contextually relevant have been endorsed [4] and evidence suggests that nutrition improvement is more likely if issues are widely understood, discussed and owned by the people and organisations for whom behaviour change is intended [5].

One way of facilitating a community collaborative effort to tackle community food security and achieve improved nutrition is to encourage the formation of multi-sector groups with representation of relevant community agencies and interests [6–8]. These collaborative efforts serve as forums to connect people with diverse talents, ideas, capacities and shared concerns [9, 10] and to utilise these to identify needs, make decisions, and set up mechanisms to effect change within community organisations and service agencies [9]. The rationale for these multi-sector groups is that through providing the conditions to enhance capacity in areas such as participatory planning and decision-making, community networking, participation and commitment, outcomes will be better realised and maintained.

Evidence however on the effectiveness of multi-sector groups in improving community-level outcomes is limited and mixed [7, 11, 12]; attention has been focused on the more intermediate benefits of multi-sector groups in fostering community capacity as important precursors to system level change. In this respect investment in achieving goals of community capacity need to run parallel to achieving program goals or health outcomes [13]. Goodman et al. [14] indicate that failure to invest in building the conditions of community capacity may prevent or attenuate program outcomes. Understanding the characteristics of community capacity and how to enhance these assets could therefore support a multi-sector group to achieve its desired outcomes.

MacLellan-Wright et al. [15], Rifkin et al. [16], Gibbon et al. [17] and Laverack [18] made valuable contributions to the evolution of capacity development models and to investigating participatory processes which actively engage communities to evaluate their capacity. Similarly to Jackson et al. [10], these authors suggest that capacity measures be developed in consultation with the community and be adapted to the uniqueness of each community and setting [10]. In the context of remote Indigenous Australia, Laverack et al. [19] applied a model of capacity development previously used in Fiji [20] to assess capacity development with health action teams in three communities. The model comprised nine capacity development constructs with adaptations made to the interpretations of these to suit the cultural setting.

Through a review of capacity development models including the model used by Laverack et al. [19], we previously described a set of capacity development constructs common to the models reviewed [21]. These constructs included a sense of community, an assets-based approach, leadership, participatory decision-making, partnerships, learning opportunities and skill development, a development pathway (including planning and evaluation processes), resource mobilisation, and communication (see Additional file 1: Table S1).

Remote Indigenous Australia is uniquely different from many other social and cultural contexts. This difference stems from a combination of factors including its recent history of European invasion, colonisation and oppression, preservation of multiple languages and retention of traditional governance structures and cultural practices despite the influences of the dominant Western worldview. There is poor understanding in the literature of the characteristics of community capacity that may affect program implementation and program outcomes in this unique context, although community ownership, community participation and leadership have been identified as important elements of successful health-related initiatives [22].

We had the opportunity to explore the characteristics of community capacity and develop a tool through the Good Food Systems, Good Food for All Project (GFS) that commenced in 2009. This project was a whole food system-focused initiative undertaken with four Indigenous communities. The project aimed overall to develop an approach through community level multi-sector food-interest groups to address food security through encouraging local organisations and groups to enhance their programs and services.

Working within this overall project, we aimed to assess the relevance and meaning of the common capacity development constructs in the context of the perspectives and worldviews of community members in remote Indigenous Australia; and through this, develop a tool for the purpose of multi-sector food-interest groups to foster capacity development. We set out to determine (i) if the literature-derived community capacity constructs were important in helping to reflect on community capacity; (ii) if the characteristics associated with each of the constructs differed from that in the literature; (iii) if there were gaps between literature- and community-derived characteristics; and, (iv) if the constructs were useful in appraising community capacity. This paper aims to describe this process.

## Method

### Study design

In seeking to develop an appropriate tool and in taking the perspective of Indigenous stakeholders with an interest in the food system, we planned our process over five steps that involved : (i) test of clarity of construct meaning; (ii) inductive derivation of community capacity constructs; (iii) application of community-derived constructs to the capacity development of multi-sector food-interest groups; (iv) a cross-check of community-derived constructs and their meanings to literature-derived constructs, and; (v) achieving consensus on tool constructs.

Data specific to this paper were collected over a three-year period (2010–2012) that involved two on-site visits to one community, and two urban-based workshops that brought together stakeholders from each of four remote Indigenous communities who had various roles in the food system. These data were augmented by meeting minutes and reports recorded from the multi-sector food-interest group meetings and workshops that occurred in each of the four communities as part of the GFS Project.

Ethics approval for all aspects of the GFS Project was attained from the Human Research Ethics Committee (HREC) of the Northern Territory Department of Health and Menzies School of Health Research (ref. HREC 09/07), Cairns and Hinterland Health Service District Ethics Committee (ref. HREC/10/QCH/71-678) and the Central Australian HREC (ref 2009.02.02). Signed partnership agreements were negotiated with participating organisations and communities.

Although here we are documenting a tool development process with community participants rather than a straightforward qualitative study involving community, we in the research team have endeavoured to adhere to the RATS guidelines [23] where applicable, in clearly documenting the relevance of the study questions, the appropriateness of the method employed, the transparency of procedures undertaken and the soundness of our interpretive approach.

### Study setting

Two of the remote Indigenous Australian communities that participated in the GFS project were situated on the North Australian coast, another was in escarpment country in Northern Australia and a fourth was in the Central Australian desert. The communities varied in size from 250 to over 2000 residents, and shared a relatively recent history of European colonisation. Multiple languages were spoken in each community with English rarely the first language. The four communities were of Aboriginal heritage and diverse in cultural practices. All were accessible only by long-distance road travel, small aircraft and/or boat. They were all serviced by limited public facilities – for example schools and health clinics – and private resources such as banking, telephone and a community store.

### Participants

In each community, stakeholders with an interest in the food system were invited to meet as part of the GFS project. In three of the four communities, an average of 10 (8–12) stakeholder meetings (including annual planning workshops and review meetings) occurred over the period of the GFS project. In the fourth community only one annual workshop and two review meetings took place. Participation in these workshops/meetings was voluntary and open to any community member (both Indigenous and non-Indigenous) or external stakeholder who had an interest in, or who might impact on, the local food system. Participants included community elders and other community members, gardeners, store managers, owners and operators (including store and take-away outlet staff), public health nutritionists, other health professionals, school, crèche, and aged-care staff, and government officials involved in food related policy and service delivery. A member of the research team (project facilitator) and a local resident Aboriginal community co-ordinator (employed by the project) facilitated these meetings.

One community was conveniently selected as the field site to test the community capacity constructs due both to its relatively close proximity to Darwin (a 700 km round trip via road) and also because the food-interest group in this community had met more frequently over the entire study period compared to the other three communities and had a relatively stable group of people attending meetings.

### Tool development process

Step 1. Test of clarity of construct meaning (March 2010, Year 2 of the GFS project).

This first step aimed to determine the relevance and meaning ascribed to each of the literature-derived capacity development constructs (as listed in the introduction and shown in Additional file 1: Table S1) from the perspective of members of the food-interest group in the field site. Eight people representing community elders, the health centre, local government, the community market garden and the store participated in the community-based food-interest group meeting. Seven of these people were Aboriginal. Members of the food-interest group were asked as a collective to firstly review each construct and its definition, to comment on its meaning and then to appraise their capacity using each of the constructs. Each construct was discussed in depth with specific and practical examples given by the facilitator to assist participants to consider the application of the construct to their situation. All constructs were considered by participants as important to community capacity development and through the appraisal process specific areas of capacity development were identified as in need of strengthening. The appraisal process took 50 minutes in total. Results from this appraisal process are shown in Additional file 1: Table S1.

In appraising the group’s capacity, the eight constructs were displayed on an A3 sized paper around an image of a coloured shooting target or bull’s eye. The purpose of the shooting target was to provide a visual scoring system for members to consider how well the food-interest group was gaining or utilising their capacity to address community level food security, and to point out where on the circles they might place themselves. In contrast to that intended, this visual aid of a shooting target was viewed by participants in the opposite way where the central circle of the target represented the construct strength as ‘just developing’ and the outer circles represented progressive steps to ‘fully developed/very strong’ , just as a heavy stone causes ripples to spread more distantly. From this the concept of a ripple with five concentric circles emerged. In subsequent activities in step 3 this concept was successfully used with a large drawing of a ripple placed in the middle of each of the participating community groups. Each of the measures were then appraised by each group in relation to their relative position from ‘just developing’ in the centre to ‘fully developed/very strong’ on the outer circle (see Additional file 2: Table S2).

Step 2. Inductive derivation of community capacity constructs (November 2011, Year 3 of the GFS project).

This step aimed to determine the most pertinent set of attributes perceived by participants as important to community capacity. Fourteen participants, from all four project communities participated in an urban-based workshop with 11 of these being Aboriginal. Two to six people represented each community. In community groups, participants were asked to identify and describe the characteristics of a ‘really strong’ food-interest group. The term ‘strong’ is a colloquial term used in remote Indigenous Australia generally used to denote the strength or resilience of an object, person or structure in relation to its purpose. These characteristics were then categorised by two of the authors (JB & CB) according to common themes and each category was given a label. These categories were then checked with the participants. The eight capacity building constructs that emerged from this exercise are presented in Additional file 2: Table S2.

Step 3. Application of community-derived constructs to the capacity development of multi-sector food-interest groups (November 2011, Year 3 of the GFS project).

To test if step 2 workshop participants could then use the community capacity constructs and “ripple tool” to appraise their community’s capacity, the four-interest groups discussed each construct amongst themselves and marked on the ripple circle where they viewed the development of each construct. Similar to the method described by Gibbon et al. [17], discussion points and reasons given by the group for the scoring of each construct were recorded. Additional file 2: Table S2 shows that the areas identified as requiring strengthening varied across communities. In the three communities where the food-interest group had met more than twice, the constructs “knowledge and skills” and “good planning” were scored relatively higher than the constructs “leadership/ right people” and “commitment to action”, which were identified as needing the most strengthening.

Later in November 2011, this process was repeated in the field with a community food-interest group. Ten people participated of whom five were Aboriginal. The process for completing the appraisal was modified from that used by McDonald et al. [24] with the role of the facilitator being to:

- Provide a verbal description of each construct based on the characteristics of the construct identified previously by workshop participants
- Ask the group to reflect and comment on how strongly they saw the construct performing in their community;
- After listening to the group’s discussion, place a card with the construct label on a circle of the ripple tool that seemed to best represent people’s comments and ask if this matched with their perception; and then,
- Manage the discussion until consensus was reached and write agreed discussion points on a sticky note and also display on the ripple;
- Take a photo for future comparison with the next community capacity appraisal.

**Figure 1:**
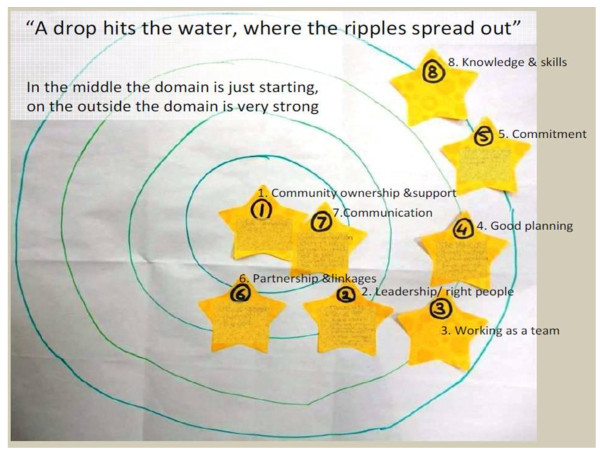
**An image showing use of the ripple by the community food group in step 3.** The reasons given by the food group for selecting a ripple for each measure were recorded on the yellow stars and stuck to the image.

Participants demonstrated an understanding of each construct and were able to relate them to their community context. Most participants engaged in the discussion and different views were expressed and taken into consideration by the group in reaching consensus on the construct score. Food-interest group members scored the constructs “knowledge and skills” and “commitment to action” as very strong/fully developed, and “community ownership and support”, and “communication” as just developing and in need of strengthening (see Figure 1). The group perceived the process of planning to be well developed, but were concerned that prioritised actions were not being implemented due to the already heavy workload of participants. Overall, participants identified that more investment was needed in gaining greater support from the wider community and encouraging participation from sectors and community groups not represented.

Step 4. A cross-check of community-derived constructs and their meanings to literature-derived constructs (February 2012, year 4 of the GFS project).

The aim of this final step was to match community-derived constructs of community capacity and their meanings to literature-derived constructs and to lastly propose a capacity appraisal model that could be used by community level multi-sector groups to consider their capacity. This step involved a review by the authors (JB, CB and SL) of all data collected from steps 1 to 3 augmented with food-interest group meeting minutes and workshop reports, and the comparing and contrasting of the capacity building characteristics and associated meanings captured in these data with the literature-derived constructs until all relevant data had been matched. Data were also checked for any new emerging constructs and/or divergence in meaning within constructs. Through this process, no new constructs to the literature-derived constructs were identified and all the literature-derived constructs were found to be represented by participant-derived constructs, except for ‘assets-based approach’ and ‘resource mobilisation’ , although elements of these were identified. Different terminologies to describe constructs were also used.

Step 5. Achieving consensus on tool measure terms (October 2012).

**Figure 2:**
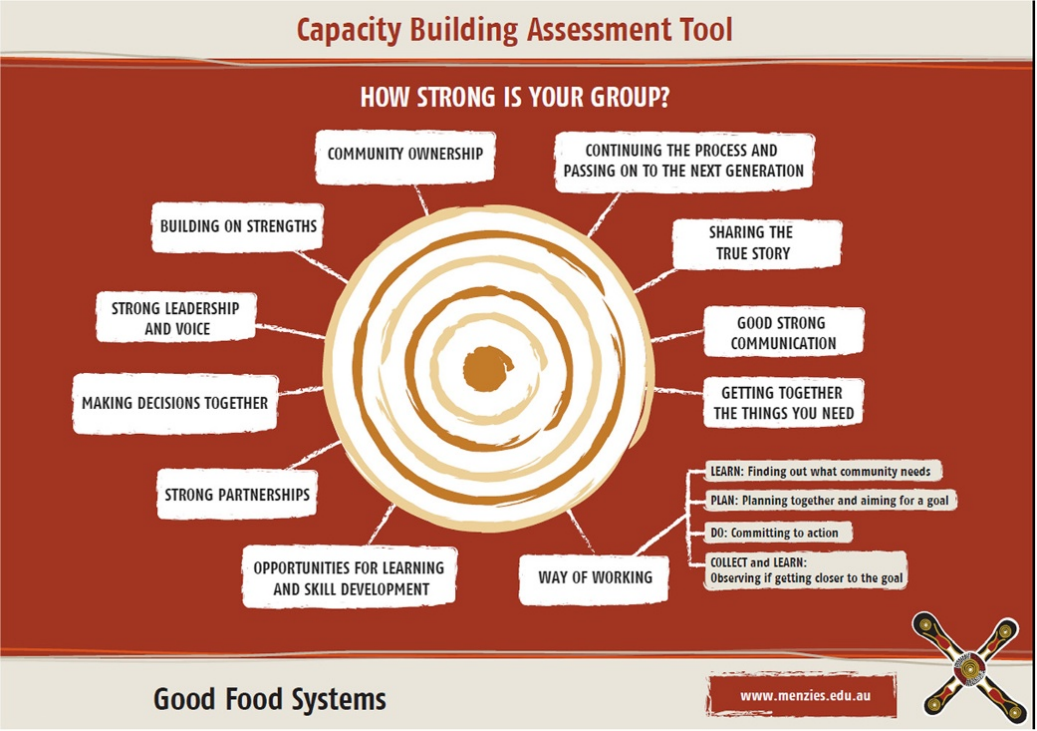
**The community capacity appraisal model.**

The literature-derived constructs were refined in a workshop with three community coordinators from two study communities and consensus was reached on the structure of the appraisal tool shown in Figure 2.

## Results

Arising from the process outlined above, this section describes the specific meanings attributed to each of the constructs, and the changes from the labelling of the literature review construct to those determined by participants.

### Sense of community → community ownership

The term ‘community ownership’ as used by participants most closely matched the literature derived construct ‘sense of community’. Only one community group specifically used the term community ownership, however other characteristics that related to the concept of community ownership were described. These were that the group: (1) recognise and support the role of elders in the community; (2) be supported by the community; and, (3) be viewed as a community-led structure, and not an imposed structure. Attaining this was said to require good communication with the community. This in turn would result in the community being receptive to the work of the group and moreover would increase community support for related policies and/or actions.

### Assets-based approach → building on strengths

When this construct was first tested with the community food-interest group, the more commonly used problem-based-approach, was considered by some participants as more appropriate than a strength-based approach to decision-making: “*we think and see the bad things, but we then think about how this can be changed to be a good thing”.* This construct was also not specifically identified by participants in step 2 however a number of references to the unique strengths of the community such as the tight social linkages in communities and the strength of people’s cultural and historical knowledge of the community were identified from all available data reviewed in step 4. This construct was therefore retained in the proposed model.

### Strong leadership- > strong leadership and voice

From the perspective of participants, strong leadership was dependent on “*having the right people at the table*” who represented the different family groups in the community and were able to advocate for community needs. Each of the four groups identified the ideal composition of a food-interest group and key partners in a similar way. Elders were central to leadership. Other leadership qualities sought were “*strong voices in the community to lobby and advertise the needs of community”,* “*influential people from the right organisations*”, “*leaders interested in the community and supported by the people that have good knowledge of food and the community*”, “*people with ideas*”, *“people that could ask elders for advice”, “people that are able to mobilise and bring people together”,* and “*people representing different areas of the community as well as the disengaged*”. In contrast to the emphasis on the qualities of a leader in the literature, participants placed emphasis on collective leadership and community representation in leadership positions. All other constructs seemed to relate to this concept of “having the right people at the table”.

### Participatory decision-making- > making decisions together

This construct was commonly referred to as working as a team. It was recognised that people often had different views and opinions and that in this diversity “*people [needed to] feel happy to voice their point of view*” and [to] “*want to discuss their views*”. Participants also referred to the importance of seeking the involvement of higher-level community governance structures, including elders, in decision-making activity and formulating of plans. This aspect of “making decisions together” also featured in the characteristics ascribed to the community ownership construct.

### Partnerships and linking with others- > strong partnerships

From the perspective of participants an important feature of this construct again concerned the composition of the group and the ability of the different stakeholders involved to function as a team, in addition to being able to work effectively with other sectors and groups in the community.

### Learning opportunities and skills development- > opportunities for learning and skills development

This construct again centred on the composition of the group where the involvement of elders was considered key as well as the involvement of relevant others who would also provide the mix of expertise needed to support the group in: addressing the issue at hand, in attending to group processes such as running meetings and mobilising resources; and, in nurturing future leaders. Participants in two of the groups specifically referred to the importance of having people involved who had knowledge of the community, culture and traditional foods: *“respecting and using knowledge of elders; they are the law for us – the carers, they are the teachers of the land” and “they are the professors*”. Two examples given of using cultural knowledge to strengthen the capacity of a food-interest group were: *“making sure traditional foods are the first option” and “use of seasons in planning”.*

### Development pathway- > way of working

Having well developed plans in place that reflected the needs of the community and demonstrated commitment to these plans were two key aspects identified that related to this construct. Commitment to action was said by participants to be facilitated by people knowing their roles and responsibilities and was demonstrated through members taking accountability for actions, helping to attract members, attending meetings, and actively communicating with the community. It was considered important that members be seen as “*action people*” and “*passionate about their community*”. A number of assessment, planning and monitoring functions were also identified that related to this construct, such as: *“know[ledge of] the community’s needs [through] use of surveys”, “strong plans with actions”, “people coming together to work together to achieve goals”, “groups learning from each other”*, a *“clear path”* where *“people know the goals”* and where *“leaders and groups* [are] *reflecting on the process and progress”.* These processes of a strong food-interest group making the sequence of a good way of working were later defined as ‘finding out what the community needs’ , ‘planning together and aiming for a goal’ , ‘committing to action’ , and ‘observing if getting closer to the goal’.

### Resource mobilisation- > getting together the things you need

This construct again related to “having the right people at the table” who could help mobilise resources and who had the skills to write grants and access funds.

### Communication and dissemination- > good communication and sharing the true story

All four groups in step 2 identified feeding back information and listening to community needs as key characteristics of a strong food-interest group. The purpose of communication was primarily to maintain good relationships across sectors and community groups. Sharing the true story was to ensure all *“stakeholders were well-informed”, “that the elders had the true story”,* were *“kept up-to-date”,* and *“that everyone knows what’s happening”.*

### Sustainability- > continuing the process and passing on to the next generation

This construct did not emerge as a characteristic of a strong food-interest group in step 2 however in workshop and meeting minutes and reports many references to the notion of sustainability were identified. The community co-ordinators elected to include this construct in the final model as they saw that an important aspect of community capacity was to continually reflect on efforts to *“keep things going”.* A community co-ordinator explained: “*We have been travelling along, if we stop now it will be like falling off a cliff, but if we can continue, it will give us the bridge to get to the other side and keep working. Without this bridge, this work just stops and we don’t get to where we are going”.*

## Discussion

This study is important because it contributes to the very limited published literature on the way in which capacity appraisal models may need to be modified to suit different contexts. We found that the constructs commonly used to appraise community capacity development were well accepted by both Indigenous and non-Indigenous people involved in planning and service provision in four remote Indigenous Australian communities and could be used to identify areas in need of strengthening. The specifics of each construct however differed from those derived from the literature yet were similar across the four communities and had particular meaning for those involved. This finding strengthens the proposition of Labonte and Laverack [25] that there are key features of capacity development that are transferable across different communities but that differences in specifics are likely to be contingent on the social and cultural context of those involved [25]. For example, we found that some elements commonly associated with the construct ‘sense of community’ in the literature [14], such as a feeling of belonging and a sense of caring and sharing among people in the community, were identified in this study, whereas recognising community history, culture, language, and issues of identity and place, also associated with sense of community in the literature [26] were not explicitly stated by participants. This by no means infers that these elements are not important in the context of remote Indigenous Australia, but that in this study they were considered in a different way. In the four study-communities, Indigenous culture and languages predominate and elders are the custodians of these to ensure their survival. Indeed a central role was ascribed to elders in six of the 11 constructs in the proposed model thus ensuring the integrity and spirit of cultural values in the functions of the food-interest group and providing the path for success. The concept of community ownership as referred to by participants in itself in many ways related to embracing cultural values and identity and ensuring cultural safety to enable full participation of community members. These differences in specifics reinforce the importance of developing particular measures of success for each construct in consultation with the community to capture the uniqueness of each community and the relevant characteristics that are important to community capacity [10].

There are very complex rules around relationships in the Indigenous Australian context that determine processes of engagement, and relationships are prioritized and viewed as the cornerstone to working effectively as a collective. A common thread to each of the constructs was the relationships between the people involved both with each other and with the wider community. It was clear that participants recognized that improving food security and nutrition in the community required the involvement of different players representing both the community groups and community services. Who exactly these people should be was a focus of each of the four community food-interest groups and often a sticking point and seen as an obstacle to making headway when it was felt that the “right people” were not yet involved. Bound in the “who”, are those who can provide the leadership, knowledge and know-how to support the group to realise its goals. This is synonymous with the view of Butterfoss ([9], p 328) that “the key asset of any coalition is its members, and the role of the coalition is to mobilise effectively members’ commitment, talents, and assets to effect change” [9]. Investing time in identifying the roles and responsibilities of the group, mapping these with key group members and how they relate among themselves, understanding the cultural governance structures of the community and keeping the rules of engagement transparent are likely to be paramount to forming a solid structure on which capacity can be further developed in the study context.

We also found that those constructs featuring strongly in other capacity development models in the literature [21] such as learning opportunities, skills development and resource mobilization were not prominent in this study. In contrast, the constructs receiving less emphasis in the literature such as community ownership, commitment to action, and communication were prominent in this study and together with leadership and strong voice, seemed to receive most attention when participants appraised their own community capacity. These constructs have previously been observed as important indicators of community capacity when used with other non-Western or marginalised populations [10, 27].

Strategies identified to strengthen these focused on actively seeking the engagement of elders and the support of the community through home visits and making the actions of the group visible to the rest of the community. These strategies were similar to those reported by Laverack et al. when assessing capacity development with Indigenous people in Northern Queensland [19]. ‘Buy-in’ and support of communities seemed pivotal to the functioning of the food-interest groups. This focus may have also been indicative of the relatively early stage of establishment that defined each group [8] and the need to set the conditions to then be effective in achieving outcomes. Ongoing dialogue with the community and demonstration of activity seemed to be a pathway to gaining community ownership and support. This is particularly salient, as the emphasis we observed that food-interest groups placed on visible activities such as cooking demonstrations or campaign days, may have been more directed to achieving the interim step of community support and effective relationship building in the establishment stage of the food-interestgroup than in solely promoting healthy eating.

Chino et al. [26] highlight that it is often wrongly assumed that members of Indigenous communities can immediately begin to resolve an issue, whereas in contrast these authors suggest robust attention needs to be paid to the processes required to build trust and achieve effective relationships and communication. However the reality is that often funding conditions and program timelines drive practices which tend to place more emphasis on achieving outcomes often to the neglect of relationship building [28]. The Indigenous people participating in this study not only had to navigate the continually changing and sometimes unfamiliar terrain of Aboriginal-non-Aboriginal relationships, but also the cultural relationships that strictly prescribe processes of communication. Similarly the non-Aboriginal participants were often finding their way in unfamiliar territory. We found time and resources and an appreciation of the complexity of social and cultural relationships were needed to support this process [26] and yet these processes in many instances are under-valued or have not been elucidated. The features of successful interventions in Aboriginal and Torres Strait Islander settings have involved local people, formed effective partnerships, trusting relationships and community support [22]. A reorientation to approaches to food security in the remote Indigenous context where community capacity goals are considered in parallel with outcome goals, or at least as genuine incremental goals along the way, may well help to lay a more solid foundation for improved practice in service delivery and program sustainability [13]. In fact, provision of adequate time to allow for these basic foundations to be established might help to address the limited observable effects of projects and programs currently experienced in remote Indigenous Australia. The community capacity appraisal model evolving from this study provides a process on how to approach this.

In this study we started with a set of literature-derived capacity development constructs and sought to assess the relevance and meaning of these from the perspectives and worldviews of community members in four Indigenous communities. This could be seen to be a limitation, since had we taken an entirely inductive approach to developing a set of constructs building on Indigenous ‘ways of knowing’ , the model may have evolved differently but with less opportunity to compare with those developed elsewhere. Step 2 did however employ an inductive process of inquiry and through this process we found commonalities between participant-derived and literature-derived constructs. The shift from target to ripple is a clear example of needing to view the world from that of the people involved. The ripple concept that was identified by participants in this study is similar to the ‘spider web’ approach for visually presenting capacity development used in Nepal and other non-Western contexts [16, 17, 29]. We found that the ripple scoring process stimulated rich dialogue around each construct. A strength of the subjective process we used in collectively appraising constructs is that it does not confine people’s consideration of the construct and allows new perspectives to emerge. To reduce subjectivity in assessing community capacity, Laverack [18] developed a process of ranking constructs [18]. As greater insight is acquired on the meaning of community capacity in the context of remote Indigenous Australia, a similar process to strengthen rigor in appraising capacity development over time, could be employed while maintaining scope for discussion and divergent views to be voiced.

At this stage, a clear limitation to assessing the relevance of literature derived constructs to a particular cultural and social context is that there is no internationally agreed set of components or standard definition of constructs from which to base an assessment, and this acts as a limit to subsequent comparison between contexts. At present, comparisons in construct characteristics across different cultural and social contexts therefore need to be made with caution as the meanings and specifics ascribed to the different constructs may differ particularly if developed in-situ.

There are other potential limitations to the proposed model from this study that could be addressed through further research. We developed this model with the involvement of four remote Indigenous communities and a diverse group of stakeholders with various roles in the food system that to some extent represents the diversity observed across communities. Community capacity however is a complex concept that has many dimensions and it is likely that we have not fully captured these – for example, the views of community capacity from the other Indigenous group in Australia, Torres Strait Islander communities, have not been captured. We tested the appraisal process in-situ with a food-interest group that had been meeting at least every three months for nearly two years. Further testing with other community groups could provide insight into the relevance of different constructs at different stages of capacity development. Whether the model when operationalized to evaluate community capacity picks up on the most important aspects of community capacity pertinent to what is needed to support improvements in food security and nutrition needs to be tested. The robustness of the constructs over time to support capacity development also need testing in addition to the reliability of the ripple scoring process with involvement of different people in the food-interest group. This later point is particularly pertinent as the composition of the food-interest group, which we found to be relatively fluid, can easily change the power balance and impact on the active engagement of different people, and of Indigenous people particularly. Research is also warranted on the benefits of assessment of capacity building over the long-term and the extent to which capacity development can be associated with positive outcomes. Further research in the area of capacity development appraisal would benefit from researchers clearly stating the specifics of constructs used for each cultural and social group for which they have been adapted and applied.

## Conclusion

In conclusion, this study contributes to the relatively limited literature on appraisal of capacity development that has been undertaken with Indigenous communities. It suggests an approach for ascribing context specific meanings to a set of capacity development constructs. The embedding of reflection on the capacity development constructs proposed in our model, in the setting up and maintenance of multi-sector groups, and a particular emphasis on the building of relationships in the early stages of establishment rather than a sole focus on health-related outcomes, can be regarded as incremental process-type outcomes and consequently have potential to contribute to stronger results in the long run. Practically, the model could be used initially to support the establishment of a multi-sector group, for example, in terms of considering the composition of the group and the relationship of the group with the community, and then later to reflect on the functioning of the group in being able to effect change. The use of the ripple tool described in this study provides an effective visual tool for the group to reflect on and track its development over time. Although the appraisal of capacity takes time and resources, we believe it appears well worthwhile to enhance the strengthening of community capacity in order to tackle complex issues such as food security that require a multi-sector approach.

## Electronic supplementary material

Additional file 1: Table S1: The literature-derived constructs, associated definition and step 1 result. (PDF 85 KB)

Additional file 2: Table S2: Community derived capacity development characteristics and construct labels compared to literature-derived constructs. (PDF 175 KB)
