# Supplementary material for: From targets to ripples: tracing the process of developing a community capacity building appraisal tool with remote Australian indigenous communities to tackle food security
Source: BMC Public Health. 2014 Sep 4;14:914. doi: 10.1186/1471-2458-14-914 (PMC4246524; doi:10.1186/1471-2458-14-914)
Supplement: Supplementary file 1 — Additional file 1: Table S1: The literature-derived constructs, associated definition and step 1 result. (PDF 85 KB) [file 12889_2014_7295_MOESM1_ESM.pdf]

Table S1. The literature-derived constructs, associated definition and step 1 result

**Literature-derived community capacity construct**	**Definition**	**Appraisal of construct by community food group (step 1)**
Sense of community	There is a strong sense of community and people wanting to work together for the benefit of the community (labeled "participation" in step 1)	The group is working well together. Everyone feels able to attend the meetings, happy to voice their points of view, and want to discuss their views.
Assets-based approach	The group acknowledges the past and builds on past and current strengths to progress and work towards achieving goals for the community.	There are lots of strengths within the group and together members have a lot of knowledge and information. However we need to talk more and share together. We think of and see the bad things, but we then think about how this can be changed to be a good thing.
Leadership	Leadership involves the ability to mobilise the community through recognising that the voice of the community needs to be heard. Leadership involves acknowledging community and individual achievements, facilitating community decision-making and collaboration, and creating a path for others to follow. This involves understanding the big picture, having the right information, knowing the track to follow and how to move forward. The development and emergence of new leaders is fostered. The right people are at the table (i.e., all community members are represented appropriately).	We have leaders within this group: community leaders and shire councillors. The two local project staff are doing a good job. We do need more skills and knowledge about the process of meetings from the non-Aboriginal perspective and how to exercise leadership in this context.
Participatory decision-making	Everyone wants to be involved and is able to be involved Participation is promoted and supported.	This was discussed under sense of community.
Partnership/linkages/net working	There is support for the group from organisations within the community, such as the school, health centre, store, shire, and organisations external to the community. These supports can be drawn from to help make improvements. Different groups support action for nutrition improvement.	We have a long way to go.

Learning opportunities and skills development.	Group members have the skills and knowledge required to achieve their goals, and are transferring their skills and knowledge into action. Skills are further developed through participation in the group.	Everyone in the group brings different skills to the group and different views; and can come together to work together to achieve goals. Members of the group can always learn more from others to help strengthen the groups capacity and continue to progress in addressing the issue. We need to know more about running a meeting and being in a committee/ or being a member of a group.
Development pathway shared vision and clear goals<br>(community needs assessment;<br>process outcome and monitoring;<br>sustainability	There is a shared vision. The group is clear on its goals and regularly reflects on progress. The group works well together to achieve outcomes. Group members understand their role within the group.	More people need to be involved – school, aged care, high school children, CDEP workers, and particularly young people to learn about meeting processes. There is a lack of communication where some people have their own views and only consider what they want to achieve. To mobilise these different groups we need to meet in the open in the different areas of the community.
Resource mobilization	The group is able to get resources needed to get the job done and make improvements.	The group has knowledge of funding and training opportunities available and is able to prepare applications. This is often about knowing what, where, how and who.
Communication (dissemination)	There is regular and timely feedback of information given within the group to support decision-making.<br>Stakeholders are well informed and kept up to date.<br>There is honest and open sharing of thoughts, ideas, and information between people where everyone is informed, takes responsibility to share and seek information, and feels safe to make comment.	A lot more work needs to be done about communication so everyone knows what is happening.
