# Supplementary material for: From targets to ripples: tracing the process of developing a community capacity building appraisal tool with remote Australian indigenous communities to tackle food security
Source: BMC Public Health. 2014 Sep 4;14:914. doi: 10.1186/1471-2458-14-914 (PMC4246524; doi:10.1186/1471-2458-14-914)
Supplement: Supplementary file 2 — Additional file 2: Table S2: Community derived capacity development characteristics and construct labels compared to literature-derived constructs. (PDF 175 KB) [file 12889_2014_7295_MOESM2_ESM.pdf]

Table S2. Community derived capacity development characteristics and construct labels compared to literature-derived constructs

**Literature-derived construct**	**Community-derived capacity development characteristics (Step 2)**	**Construct label (Step 2)**	**Application of community derived constructs to capacity development of food groups (Step 3)***
Sense of community (commitment to action)	The community is interested in the work of the group and cares about the groups work.<br>Community has ownership of the group.<br>There is support from the community in order to do a good job.<br>Group members are selected by the community.<br>Group members listen to the whole community.<br>Members of the group ensure that traditional food is the first option and that foods important to people for cultural reasons are available.	Community ownership and support	People not interested- no help (where stone hits the water);<br>need more support from community (2);<br>Just starting (where stone hits the water);<br>have strong community support (5).
Assets-based approach	-	-	
Leadership	The right people are at the table who are nominated by the community, who represent the different traditional groups, including the traditional owners, and are strong and sure of what they can do. This includes: Influential people from the right organisations; different people who are strong, speak out, are observant and supportive and represent different interest areas including community nutrition workers or Aboriginal health workers; and to include members who are motivated due to having had a personal experience with sickness; the group is able to advocate and put pressure on where needed to facilitate change.	Leadership/ right people	More people to help with ideas, need the right people (1); need other agencies on board (1); just starting (where stone hits the water); need to get young community members involved (3).
Participatory decision-making	People can work together as a team.<br>Members of the group really know each other and get on with each other.<br>Everyone involved in services or organisations	Working as a team	Too many jobs for the number of people (1); we work together and know each other – we are flexible with time and give support (3); had good community

	relating to the supply and access to food should be there (including community representatives and the groups that are hard-to-reach).		input at the workshop, but just starting (where stone hits the water); community is getting involved with health (4).
Partnership/linkages/ networking	The group is linking and working with people who can help, such as nutritionists and health workers.<br>The group is working together with the store management structure and the store manager.<br>Main organisations and key community members are involved and taking responsibility.	Partnerships and linkages	Building a new shop, but still need a strong store committee to link with (3); now we are seeing partnerships being formed (3); we have commitment to partnerships (1); we have some good partnerships between the different agencies (2).
Learning opportunities and skills development	People in the group have knowledge and skills: including good knowledge of food, the community; and cultural knowledge (including knowledge of traditional foods).	Knowledge and skills	Have good understanding of community needs (5); leaders in the community and the assistant store manager involved and feel we have the right mix of knowledge (3); we are currently doing training for new members (2); there is knowledge and skills in hunting, caring, teaching and work support (5).
Development pathway shared vision and clear goals<br>(community needs assessment;<br>process outcome and monitoring;<br>sustainability	Good planning.<br>Commitment to action, and commitment to attend meetings.<br>The group develops strong plans with actions.<br>Group members:<br>- take accountability for actions that relate to their area of work and are 'action people';<br>- are committed to actions<br>- are passionate about their community and care<br>- regularly meet together	Good planning; Commitment to action	**Good planning:** Been having meetings for a long time and so we do this well (3); starting<br>good planning but need to go out to community into action (2); the workshop helped with planning (1); we have all the different agencies participating in planning (5)<br><br>**Commitment:** Need more support from community members (2); same as planning (2); we have commitment, but are seeking more support (1); sorry meetings and royalties can impact on

			this(2).
Resource mobilization	-	-	
Communication (dissemination)	Members of the group are visiting people in the community and sitting with them.<br>There is good communication between members of the group, with the community store and with other stakeholders.<br>The group is delivering the message to the community and keeping the community informed of plans and progress.	Communication	Could be better/more feedback from store/committee (1); still need to feedback to store board, reference group and to other groups to get them involved (2); we need community input through a survey (2); we communicate well through calling community meetings (4).

*the number in brackets or the wording 'where stone hits the water' after each comment denotes the strength accorded to the construct as perceived by each community group. The score is presented in the order of community A, B, C and D for each construct, where the food group in community A had met 12 times in total and for the other communities, 8, 3 and 10 times respectively over the entire good food systems: good food for all project period.
